# A Complex of Equine Lysozyme and Oleic Acid with Bactericidal Activity against *Streptococcus pneumoniae*

**DOI:** 10.1371/journal.pone.0080649

**Published:** 2013-11-18

**Authors:** Emily A. Clementi, Kristina R. Wilhelm, Jürgen Schleucher, Ludmilla A. Morozova-Roche, Anders P. Hakansson

**Affiliations:** 1 Department of Microbiology and Immunology, University at Buffalo (SUNY), Buffalo, New York, United States of America; 2 Department of Medical Biochemistry and Biophysics, Umeå University, Umeå, Sweden; 3 The Witebsky Center for Microbial Pathogenesis and Immunology, Buffalo, New York, United States of America; 4 The New York Center of Excellence for Bioinformatics and Life Sciences, University at Buffalo (SUNY), Buffalo, New York, United States of America; Instituto de Biociencias - Universidade de São Paulo, Brazil

## Abstract

HAMLET and ELOA are complexes consisting of oleic acid and two homologous, yet functionally different, proteins with cytotoxic activities against mammalian cells, with HAMLET showing higher tumor cells specificity, possibly due to the difference in propensity for oleic acid binding, as HAMLET binds 5-8 oleic acid molecules per protein molecule and ELOA binds 11-48 oleic acids. HAMLET has been shown to possess bactericidal activity against a number of bacterial species, particularly those with a respiratory tropism, with *Streptococcus pneumoniae* displaying the greatest degree of sensitivity. We show here that ELOA also displays bactericidal activity against pneumococci, which at lower concentrations shows mechanistic similarities to HAMLET’s bactericidal activity. ELOA binds to *S. pneumoniae* and causes perturbations of the plasma membrane, including depolarization and subsequent rupture, and activates an influx of calcium into the cells. Selective inhibition of calcium channels and sodium/calcium exchange activity significantly diminished ELOA’s bactericidal activity, similar to what we have observed with HAMLET. Finally, ELOA-induced death was also accompanied by DNA fragmentation into high molecular weight fragments – an apoptosis-like morphological phenotype that is seen during HAMLET-induced death. Thus, in contrast to different mechanisms of eukaryote cell death induced by ELOA and HAMLET, these complexes are characterized by rather similar activities towards bacteria. Although the majority of these events could be mimicked using oleic acid alone, the concentrations of oleic acid required were significantly higher than those present in the ELOA complex, and for some assays, the results were not identical between oleic acid alone and the ELOA complex. This indicates that the lipid, as a common denominator in both complexes, is an important component for the complexes’ bactericidal activities, while the proteins are required both to solubilize and/or present the lipid at the bacterial membrane and likely to confer other and separate functions during the bacterial death.

## Introduction

Most often, proteins adopt native conformations to fulfill their biological functions. Failure of protein folding can lead to cell pathology due to amyloid formation based on assembly of partially unfolded proteins, which causes a number of human ailments referred to as protein misfolding diseases [1]. These include Alzheimer’s disease, Parkinson’s disease, systemic amyloidoses, and type II diabetes. The milk protein alpha-lactalbumin in complex with oleic acid (HAMLET) is a pioneering example of a protein where partial unfolding forms a basis for functional diversity [2]. While natively folded alpha-lactalbumin (ALA) associates with galactosyl transferase in the mammary epithelium to produce lactose [3], partially unfolded alpha-lactalbumin can be stabilized with oleic and linoleic acids from human milk (and bovine, to a less extent) to form the HAMLET and BAMLET complexes (human and bovine alpha-lactalbumins made lethal to tumor cells), which have attracted significant attention due to their apparent therapeutic properties and ability to selectively kill tumor cells [2,4,5]. 

The tumoricidal activity was first discovered in casein, obtained after low pH precipitation of human milk [6], and was named MAL (multimeric alpha-lactalbumin), due to its oligomeric nature on SDS-PAGE [6,7]. Since then, many studies have been published regarding complexes between alpha-lactalbumins or other partially unfolded proteins and oleic acid (OA) that have been produced by various methods and shown to contain diverse ratios of protein to oleic acid, with cell cytotoxicity displaying different degrees of specificity towards tumor cells [8-14]. The fact that partially unfolded ALA requires oleic acid association to kill tumor cells [5] and that several proteins can complex with OA to produce higher cytotoxicity than the equivalent OA concentration in free form supports the idea that OA is required for optimal cytotoxic activity and that the protein acts, at least to some degree, to effectively solubilize and/or to optimally deliver the lipid to target cells [10,13-16]. However, the various cytotoxic mechanisms described to date also point to different and important roles for the proteins in this process. Thus, it was suggested that the ability to form stable complexes with lipids may be a generic feature of the polypeptide chain, although specific structural and functional features may vary between different proteins [17].

Serendipitously, HAMLET was also shown to confer bactericidal activity *in vitro* [18], but this effect has not been explored to same extent as its tumoricidal activity. Analysis of the antibacterial spectrum of HAMLET revealed a greater degree of sensitivity displayed by streptococci [18], gram-positive bacteria that are common causes of upper respiratory tract infections. Using *S. pneumoniae* as a model organism to characterize the mechanism of HAMLET-induced bacterial death, we recently showed that HAMLET-treated bacteria display mechanistic and morphological similarities with eukaryotic apoptosis, including cell shrinkage, DNA condensation, and DNA degradation into high molecular weight (HMW) fragments [19]. Further exploration of this mechanism revealed that HAMLET perturbs the membrane of the pneumococcus and requires specific ion transport, particularly sodium-dependent calcium influx, to initiate its pathway of death, which appears to also overlap with the physiological programmed cell death pathway(s) that already exist in the bacteria [20].

As HAMLET is the only protein/oleic acid complex, whose bactericidal activity has been comprehensively analyzed, in this study we characterized the bactericidal activity of equine lysozyme (EL) in complex with OA (ELOA) and compared it to the activity of OA alone. As HAMLET targets bacterial cells by perturbing their plasma membranes, we examined if ELOA could induce similar activity. We found that ELOA induced cell death with features similar to HAMLET, and that OA alone could mimic many of these features, but only at higher concentrations than those present in the ELOA complex, and with variable kinetics and specificity in some assays. Altogether, these results detail a second bactericidal protein-lipid complex that activates an apoptosis-like pathway of death in the pneumococcus, while also suggesting specific contributions for the protein and lipid components of the ELOA complex. 

## Materials and Methods

### ELOA production and characterization

EL was purified and ELOA was produced as described previously [11]. Specifically, a DEAE-FF Sepharose column (Amersham Biosciences, Piscataway, NJ) was conditioned with oleic acid, and equine holo-lysozyme (10 mM Tris) was subsequently added. The protein-lipid complex (ELOA) was eluted with a linear salt gradient consisting of 0-1.5 M NaCl (pH 9.0). All experiments in this study were performed using a single batch of ELOA. ELOA was characterized by NMR in order to determine the ratio of protein to oleic acid by comparing the integrals of proton signals corresponding to the protein aromatic residues to oleic acid signals as described previously [18]. The stoichiometry was determined to be 1 mole of EL per 35 moles of OA, which means that the ELOA complex contains 59% protein and 41% lipid per weight. 

### Bacterial strains and growth conditions

*Streptococcus pneumoniae* strains D39 (wild-type) [21], D39∆*lytA* (D39 without the major autolysin LytA) [22], and R36A (an unencapsulated derivative of the wild-type strain D39) [21] were used in this study. While all of these strains displayed equal sensitivity to ELOA (data not shown), we employed the LytA mutant for almost all of our studies in order to limit the autofluorescence and other potentially interfering effects generated upon activation of the autolysin. This also enabled us to measure the rupture of the membrane stimulated by ELOA alone. *Haemophilus influenzae* 2019 [23], *Escherichia coli* JM109 [24], and *Staphylococcus aureus* strain I7 [20] were also used in this study. Bacteria were prepared as previously described [18,20]. Briefly, all stocks were allowed to grow to late logarithmic phase (Abs_600nm_ of approximately 0.65), and washed twice and resuspended in 1X phosphate-buffered saline (PBS; pH 7.2; GIBCO, Life Technologies, Grand Island, NY, USA) prior to use in all assays.

### Bacterial viability assay

Bacterial viability was assessed as previously described [20]. Oleic acid (Sigma-Aldrich, St. Louis, MO, USA) was reconstituted in ethanol, sonicated to prevent micelle formation, diluted in PBS to appropriate concentrations, and sonicated again immediately prior to use in the assays. After pneumococcal incubation at 37°C in the presence of reagent, EL, OA, ELOA, and/or inhibitors for 1 hour, the bacteria were serially diluted in PBS and 100 µl of 10-fold dilution series was plated onto solid media composed of Tryptic Soy Broth (Bacto, BD Diagnostics, Franklin Lakes, NJ) supplemented with 5% defibrinated sheep blood (Bio Link Inc., Liverpool, NY). After overnight incubation at 37°C, colony forming units (CFUs) were enumerated as a measure of bacterial viability. 

### Binding of ELOA to bacteria

EL and ELOA complex were directly labeled with AlexaFluor™ 488 using a conjugation kit (Molecular Probes, Life Technologies, Eugene, OR, USA), according to manufacturer instructions. These preparations were then added to 100 µL samples of pneumococci, incubated at 37°C for 30 min, washed in PBS, counterstained with 300 nM DAPI, and visualized by confocal microscopy at 630 times magnification. Binding of AlexaFluor™ 488 was pseudo-colored green and staining with DAPI, although visually emitting blue light, was pseudo-colored red to make it easier to potentially observe co-localization in yellow. 

### Membrane potential and integrity

To detect membrane potential and rupture of the pneumococcal membrane, respectively, 500 nM DiBAC_4_(3) (bis-(1,3-dibutylbarbituric acid) trimethine oxonol; Molecular Probes) and 20 µg/mL propidium iodide (Sigma-Aldrich) were added to washed pneumococci. In a 96-well plate, a 200 µL volume of this bacterial suspension was added to each well. The plate was then placed immediately into a pre-warmed (37°C) Synergy 2 Multi-Mode Microplate Reader (BioTek, Winooski, VT, USA), where fluorescence readings from DiBAC_4_(3) (485/20 nm excitation, 528/20 nm emission) and PI (530/25 nm excitation, 590/35 nm emission) were taken every minute for forty minutes to allow for equilibration of the dyes over the membrane. After this time elapsed, the specified concentrations of ELOA or OA were added and readings were taken for the next hour. When the specific ion transport inhibitory compounds Ruthenium red (RuR; aqueous), amiloride hydrochloride hydrate (Amil; DMSO), and 3’4’-dichlorobenzamil hydrochloride (DCB; DMSO) (all from Sigma-Aldrich) were being studied, they were introduced immediately prior to the addition of ELOA or OA concentrations that were known to kill three log_10_ of pneumococci. The degree of depolarization in the presence of inhibitors was calculated as previously described [20], and expressed as a percentage of the value induced by ELOA or OA alone.

### ^45^Ca^2+^ transport assays

Calcium uptake was assessed as previously described [20]. Briefly, late log phase pneumococci were suspended in 1X PBS containing 0.5 mM CaCl_2_ (CaPBS). ^45^CaCl_2_ (PerkinElmer; Waltham, MA, USA) was added to the cells at a final concentration of 5 µCi/mL with two minutes equilibration time, and subsequent baseline measurement. The sample was divided and ELOA was added to one of the tubes, with ^45^Ca^2+^ uptake subsequently measured at various time-points by: collecting the bacteria on a 0.45 µm filter, washing the filter with CaPBS to avoid nonspecific interactions of ^45^Ca^2+^ with the surface of the bacteria, and detecting beta-counts (CPM) on a Wallac 1409 liquid scintillation counter (Wallac Oy, Turku, Finland). 

### Fluorescent detection of Ca^2+^ transport

Fluorescent detection of changes in intracellular Ca^2+^ was achieved utilizing the calcium-sensitive indicator dye Fura-2/AM (Molecular Probes), as previously described [20]. Briefly, late log phase R36A pneumococci were incubated with 5 µM Fura-2/AM for 2 h to load the bacteria and subsequently washed twice and resuspended with PBS. 200 µl of the washed/loaded bacteria were added per well of a 96-well plate, which was placed in the pre-warmed (37°C) BioTek Synergy 2 plate reader. A baseline reading was taken by measuring the fluorescence every second for 1 min (340/11 and 380/20 excitation, 508/20 emission). At the end of the baseline reading, OA was added and fluorescence readings were immediately taken every sec for another 4 min. The calcium ionophore Ionomycin (Sigma-Aldrich) was used as a positive control for Ca^2+^ transport.

### DNA fragmentation

Fragmentation was assessed as previously described [19]. Briefly, treated wild-type D39 bacteria (approximately 5 x 10^8^ CFUs) were pelleted and resuspended in 1 volume of plug buffer (150 mM NaCl, 5 mM MgCl_2_, 2 mM EGTA, 2 mM KH_2_PO_4_, pH 6.8) and 1 volume of 40°C low-melting agarose (1.0%). This suspension was pipetted into a 100 µL plug mold and left on ice to form a gel (2 plugs per sample). Each gel plug was transferred into a well of a 6-well microtiter plate (Falcon, BD Diagnostics) containing 2 mL of lauroyl sarcosine buffer (10 mM NaCl, 1% Na-lauroyl sarcosine, 10 mM Tris-Cl and 25 mM EDTA, pH 9.0) with 100 µg/ml of proteinase K and incubated for 24 hours at 50°C. The plugs were washed twice (30 minutes each time) in TE buffer (10 mM Tris-Cl, 1 mM EDTA, pH 8.0) at 4°C, and half a plug was inserted into each well of a precast 1% agarose gel (in 0.5X TBE buffer) The gel was separated at 130 V for 45.5 hours in a Bio-Rad CHEF DRII pulse-field gel electrophoresis apparatus (Bio-Rad, Hercules, CA) using initial A-time of 5 sec and final A-time of 120 sec.

## Results

### ELOA and OA induces concentration-dependent death in *S. pneumoniae*

**Figure 1:**
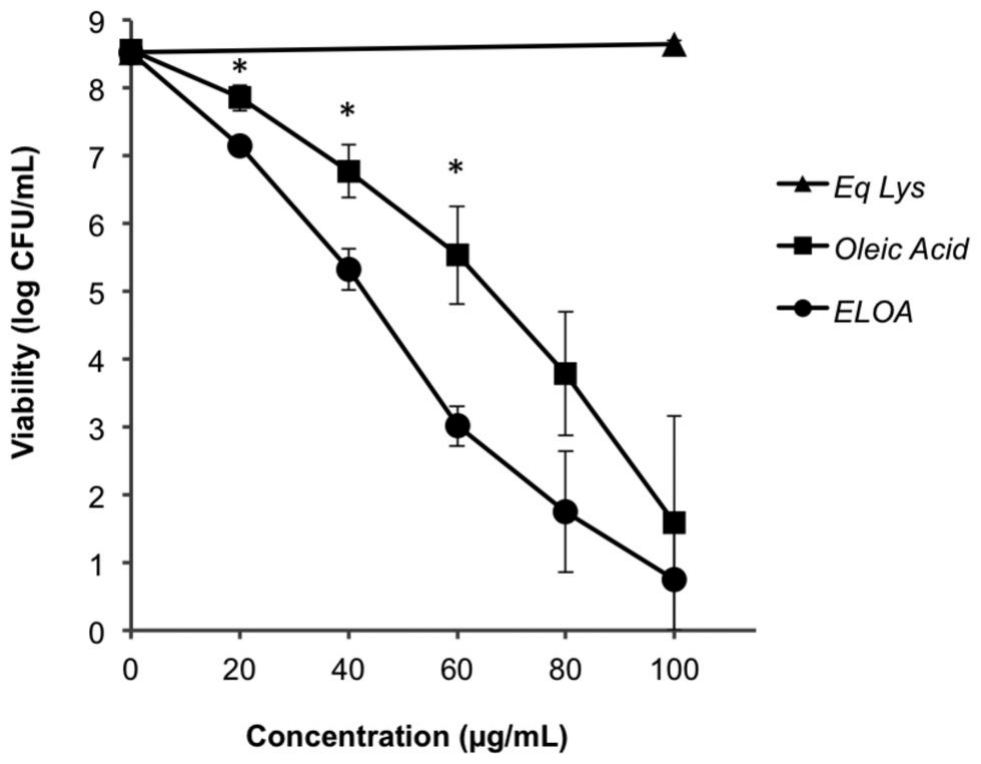
Bactericidal activity against *S. pneumoniae*. LytA-negative D39 pneumococci (D39∆*lytA*) were incubated in the presence of various concentrations of ELOA for 1 hour at 37°C. In parallel, the corresponding concentrations of EL or OA present in each concentration of ELOA were added to pneumococci to assess their direct contribution to the bactericidal activity, with the EL and OA concentrations plotted not at their actual concentrations used, but at their corresponding ELOA concentration. Bacterial viability was assessed and presented as colony-forming units (CFU) per mL of bacterial suspension, and represents the mean of three separate experiments, with error bars representing the Standard Error. Significance was calculated using the paired *t* test with a 95% confidence interval (* = *P* < 0.05).

To first determine if ELOA was bactericidal (like HAMLET), we introduced various concentrations of ELOA into broth cultures of *S. pneumoniae*, incubated the samples for one hour, and plated them for viable counts. As shown in Figure **1**, pneumococci died in the presence of ELOA in a concentration-dependent manner. We also assessed the effect of ELOA’s individual components by adding just the protein (EL) alone or the lipid (OA) alone at concentrations that correlated to those found at each concentration of ELOA tested. While addition of EL did not display any lethal activity (Figure **1**), consistent with the resistance of pneumococci to lysozyme [25,26], OA killed pneumococci in a concentration-dependent manner (Figure **1**), which is consistent with the known bactericidal activity of oleic acid [27-30]. However, the pneumococcal death observed in the presence of the concentration of OA corresponding to each ELOA concentration (41% w/w OA content) was significantly less pronounced than the death induced by the full ELOA complex (Figure **1**), showing that ELOA kills the bacteria more efficiently than OA alone. Furthermore, approximately half the concentration of ELOA compared with HAMLET was required to kill the same number of *S. pneumoniae* cells, which could be related both to the difference in protein moieties and the higher content of OA in the ELOA complex. 

To further compare the bactericidal activity between HAMLET and ELOA, we tested the activity of ELOA (125 µg/mL) against other gram-positive and gram-negative bacterial species such as *Escherichia coli, Staphylococcus aureus, and Haemophilus influenzae*. Among them, only *H. influenzae* was somewhat susceptible to ELOA (data not shown), but at a significantly higher concentration (125 µg/mL was required to yield one log_10_ of death) than that required to kill *S. pneumoniae*. These results are consistent with the bacterial susceptibility profile for HAMLET [18]. As *S. pneumoniae* was the species with the greatest sensitivity to ELOA, it was used for all subsequent assays described here to characterize the mechanism of the bactericidal activity.

### Binding to the surface of pneumococci

**Figure 2:**
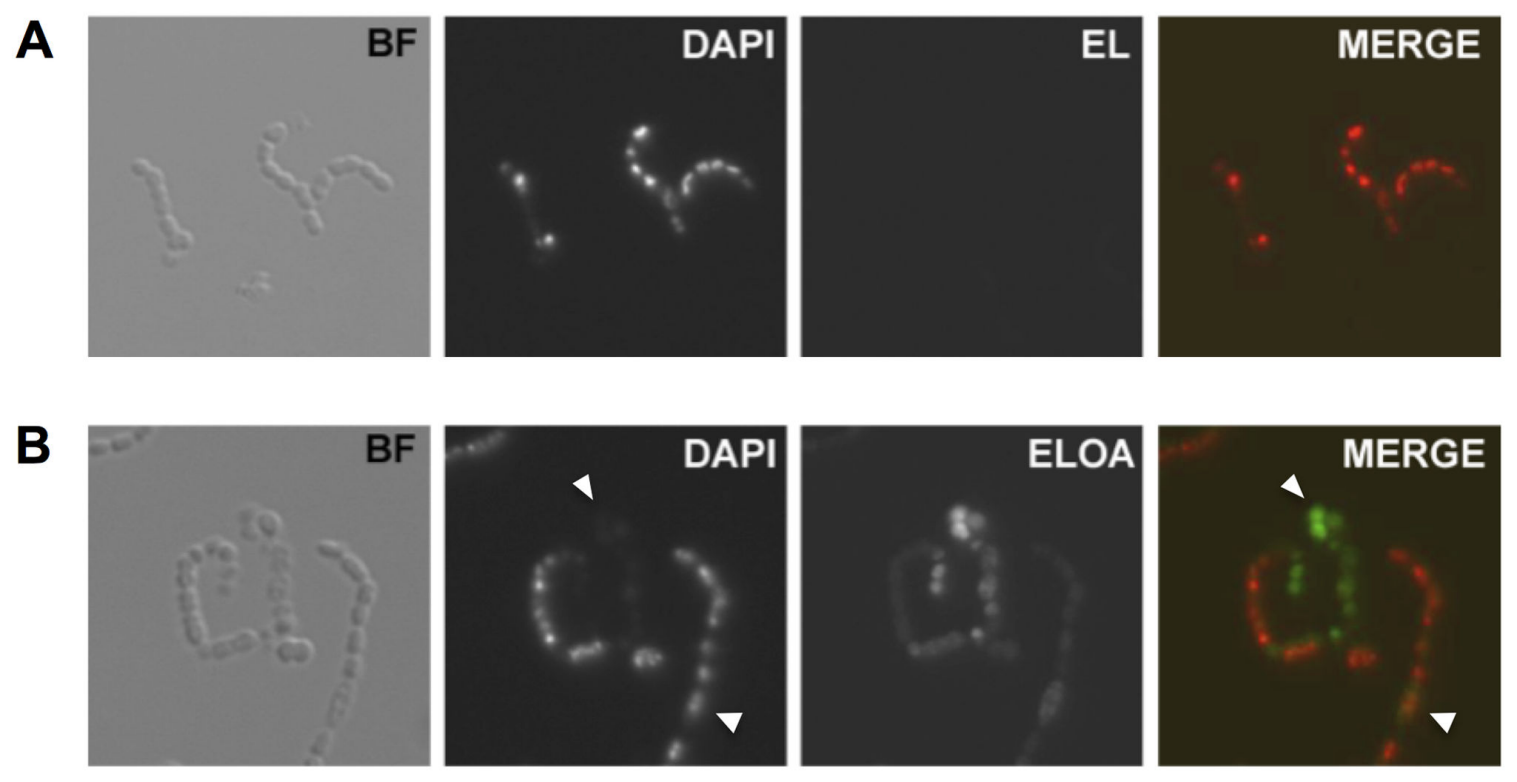
The ELOA complex binds to the surface of *S. pneumoniae*. Alexa Fluor 488 was conjugated to lysozyme (**A**) by itself and (**B**) in the ELOA complex, and incubated with D39∆*lytA* pneumococci for 20 min at 37°C. Bacteria were also incubated with DAPI to stain intact DNA, and bacteria were examined using confocal microscopy: bright field (*panel 1*), DAPI (*panel 2*), AF488 (*panel 3*), and a merge of panels 2 and 3 (panel *4*; pseudo-colored red fluorescence for DAPI, and green fluorescence for AF488 on ELOA; yellow fluorescence where staining co-localizes). Upper arrowhead points to cells that have lysed and lost their DAPI staining, while the lower arrowhead points to intact cells where DAPI staining remains and ELOA is also bound.

To investigate the interaction of ELOA with pneumococci, we examined the direct interaction of ELOA and EL with the pneumococcal surface using Alexa Fluor 488-conjugated protein. Conjugated ELOA retained full bactericidal activity similar to unconjugated ELOA (data not shown). No binding to bacteria was detected above background staining by microscopy using any concentration of EL within the studied range (1-100 µg/ml) (Figure **2A**), nor with sub-lethal concentrations of ELOA (less than 10 µg/ml; not shown). At lethal concentrations of ELOA (above 20 µg/ml), however, we observed association with the bacteria within 20 minutes of incubation (Figure **2B**, panel *3*; green fluorescence in the merged image). A homogenous interaction of ELOA with the bacterial membrane was observed throughout the bacterial population of intact cells stained with DAPI (Figure **2B**; panel *4*; green staining in the merged image). For those cells that displayed a loss of intracellular DNA staining visualized by DAPI (Figure **2B**; panel *2*; pseudo-colored red in the merged image), due to rupture of the bacterial membrane, ELOA accumulated inside the cells showing stronger total fluorescence, in a fashion not unlike the accumulation of HAMLET in tumor cell nuclei [8]. These results suggest that rupture of the membrane and death by ELOA ensues quite rapidly upon protein binding, similar to what is observed in tumor cells [11].

### The polarity and integrity of the pneumococcal membrane is disrupted

**Figure 3:**
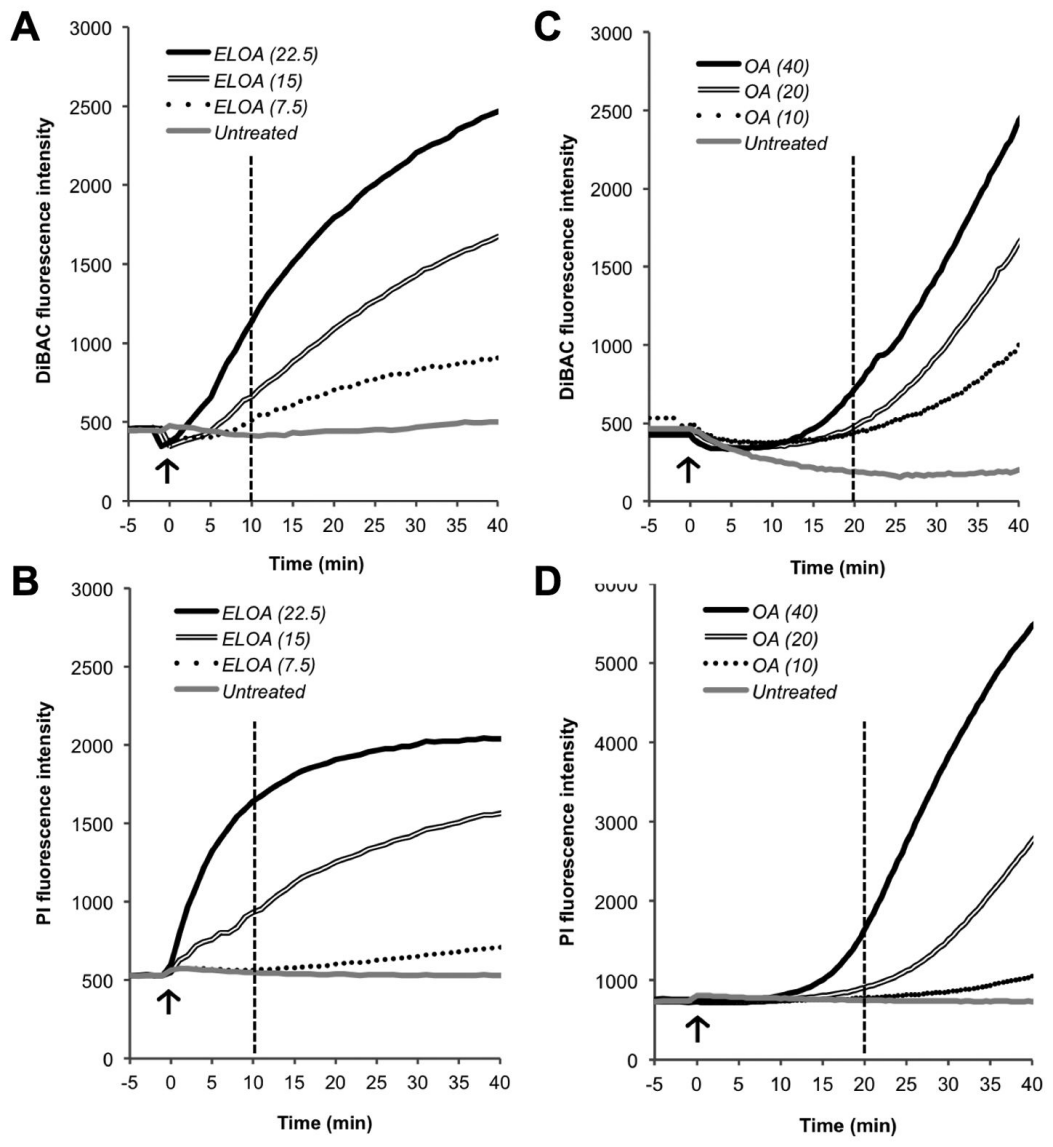
ELOA and OA disrupt pneumococcal membrane polarity and integrity. Mid-log phase pneumococci (D39∆*lytA*) were incubated with the fluorescent indicator dyes DiBAC_4_(3) and propidium iodide (PI) concurrently to detect (**A**, **C**) membrane depolarization and (**B**, **D**) rupture, respectively. ELOA (7.5-22.5 µg/ml; **A, B**) or OA (10-40 µg/ml; **C, D**) was added at time zero (*arrow*), and fluorescence was monitored over time. Depolarization and rupture graphs for ELOA (A and B) and OA (C and D) are placed underneath each other and a vertical dotted line is placed at 10 minutes and 20 minutes, respectively, to better appreciate the correlation between the two phenomena. Results presented are from a representative experiment.

Membrane potential has been shown to be involved in many essential bacterial processes such as ATP generation, bacterial autolysis, glucose transport, chemotaxis, and survival at low pH, as well as in eukaryotic processes including depolarization of the mitochondrial membrane during apoptosis [31]. HAMLET perturbs the pneumococcal membrane by dissipating its potential and subsequently disrupting its integrity. Although the depolarization event *per se* was found to be insufficient for bactericidal induction, it coincided with death and was a good marker for membrane perturbation in our assays with HAMLET [20]. As ELOA bound to the surface of *S. pneumoniae*, we sought to determine the potential effects it elicits on the pneumococcal membrane upon binding. Using the membrane potential-sensitive fluorescent dye DiBAC_4_(3), we detected a rapid and concentration-dependent depolarization of the membrane after treatment with ELOA (Figure **3A**).

Concurrently, we monitored membrane integrity using the membrane-impermeable fluorescent dye propidium iodide (PI), which fluoresces upon binding to DNA, but will only be able to enter a cell if the membrane is disrupted. Upon addition of ELOA, we observed that, like depolarization, fluorescence of PI increased in a dose-dependent manner (Figure **3B**), demonstrating that ELOA disrupts the integrity of the pneumococcal membrane. At the lowest ELOA-concentration (7.5 µg/mL), we observed that about 5-10 minutes after induction of depolarization, the PI fluorescence began to rise, indicating membrane rupture (Figure **3B**), and continued to rise throughout the rest of the incubation period. At higher concentrations the temporal difference between induction of membrane depolarization and rupture was decreased or indistinguishable, indicating that membrane depolarization and rupture occurs rapidly following the binding of ELOA to the membrane. This is different from what we have observed with HAMLET, where depolarization occurs before rupture begins at both low and high HAMLET-concentrations [20], suggesting that high concentrations of ELOA exert a different kinetics of depolarization and rupture of the membrane. 

Interestingly, upon testing ELOA’s components, we observed that the addition of EL alone triggered a low-grade depolarization but no rupture (data not shown), which is consistent with its inability to kill pneumococci. However, the effect of OA alone was different. Adding the concentration of OA (10 µg/ml) present in the highest concentration of ELOA (22.5 µg/ml) caused significantly lower depolarization (Figure **3C**) and rupture (Figure **3D**) than the ELOA complex, and depolarization and rupture started after 20 minutes of treatment rather than immediately after treatment without any temporal difference. Lower concentrations of OA showed insignificant depolarization or rupture. Higher concentrations of OA (at least 4-fold higher than that present in the ELOA complex) were required to induce comparable increases in membrane depolarization and rupture. However, irrespective of OA concentration and in contrast to ELOA, prolonged delays were observed before the OA-induced fluorescence of either dye began to rise, resulting in very different kinetic patterns and demonstrating that the ability of OA to perturb the membrane is inferior to that of the ELOA complex. 

### Pneumococcal uptake of calcium is triggered upon exposure to ELOA and OA

**Figure 4:**
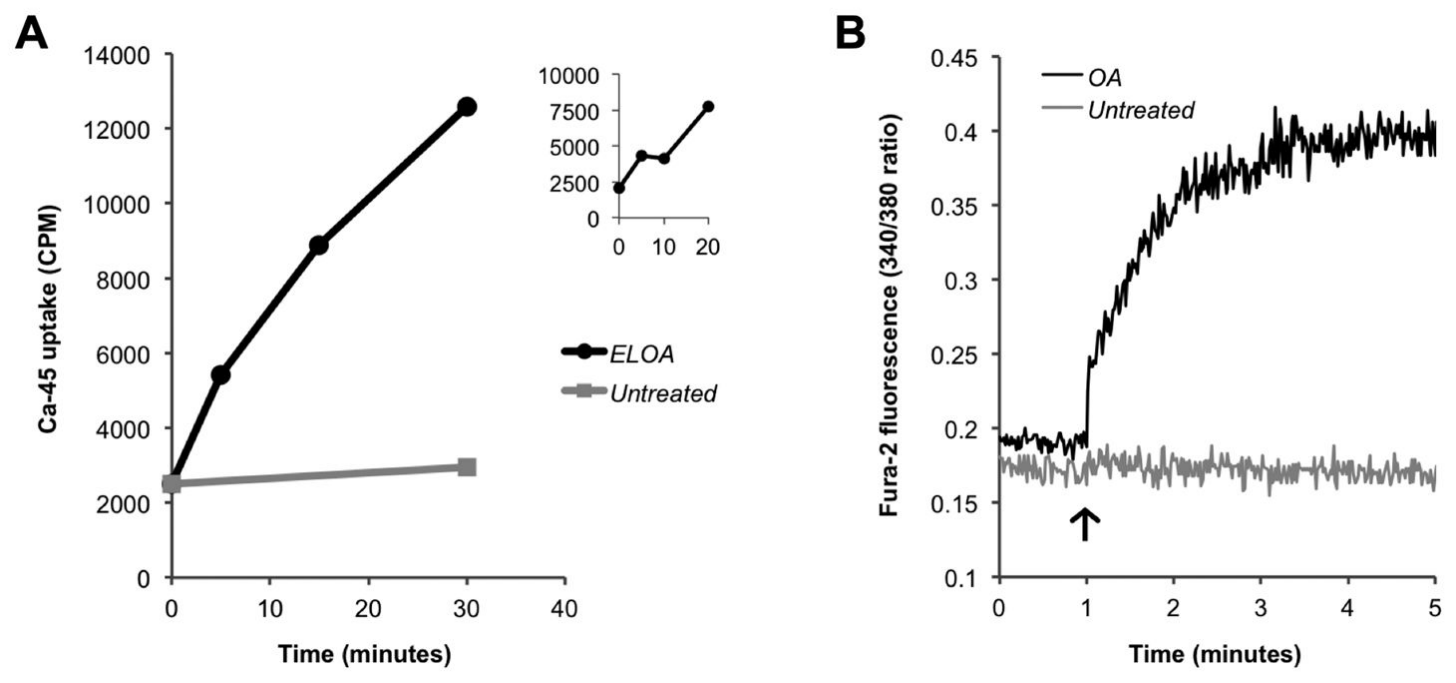
An uptake of calcium is triggered upon exposure to ELOA and OA. (**A**) Mid-log phase D39∆*lytA* pneumococci were incubated with the radioisotope ^45^Ca^2+^ (5 µCi/mL). After recording a baseline reading, PBS (untreated; *gray* *line*) or ELOA (15 µg/mL; *black* *line*) was added to the bacteria and samples were removed over time to measure the associated radioactivity (CPM). Results presented are from a representative experiment. *Inset*: ELOA-induced ^45^Ca^2+^ uptake was recorded over 10 minutes, demonstrating the early plateau in calcium uptake. (**B**) R36A pneumococci were loaded with the Ca^2+^-sensitive dye Fura-2, and after recording baseline readings, PBS (untreated; *gray* *line*) or OA (20 µg/mL; *black* *line*) was added (*arrow*) to the bacteria and fluorescence was measured over time. Results from a representative experiment are shown.

As HAMLET was shown to induce the uptake of calcium in *S. pneumoniae* [20], a feature specifically required for HAMLET’s bactericidal activity, we predicted that ELOA would induce the same effect. To test this hypothesis, we incubated pneumococci with the radioisotope ^45^Ca^2+^. Upon addition of ELOA, the levels of radioisotope inside the bacteria rapidly rose over time (Figure **4A**), confirming that ELOA stimulates the uptake of calcium into bacteria over time in a sustained manner. Interestingly, around ten minutes following ELOA addition, there was a brief “plateau” (Figure **4A**, *inset*) of ^45^Ca^2+^ levels – a brief stasis or even slight decrease in the level of the radioisotope, followed by a steady subsequent increase. This event was consistently observed in all experiments, and is suggestive of a carrier-mediated transport mechanism, such as an exchanger. Importantly, this “plateau” was also observed during HAMLET-induced uptake of ^45^Ca^2+^ [20]. 

 To evaluate the impact of OA alone on intracellular calcium levels, we used the Ca^2+^-sensitive intracellular fluorescent indicator dye Fura-2/AM. Unencapsulated R36A pneumococci were used in these experiments to increase the efficiency of dye loading. This strain shows a similar sensitivity to death and ^45^Ca^2+^ uptake in response to HAMLET [20]. After dye loading and taking a brief baseline measurement, concentrations of OA equivalent to those present in the ELOA experiments above showed little detectable increase in intracellular calcium concentration (not shown). Rather, three times higher concentration of OA than present in the ELOA complex was required to observe an increase in the fluorescence signal (Figure **4B**), demonstrating that OA alone, at higher concentrations, is capable of triggering an uptake of Ca^2+^ into the pneumococcus. 

### Inhibition of ion transport limits the bactericidal activity

The effect of different ion transport inhibitors on membrane depolarization and death has been demonstrated for HAMLET [20]. Those studies showed that inhibition of Ca^2+^ transport with the Ca^2+^ channel inhibitor ruthenium red (RuR) and the Na^+^/Ca^2+^ exchange (NCX) inhibitor 3’,4’-dichlorobenzamil (DCB), effectively limited HAMLET-induced depolarization and death. Inhibition of other ion transport mechanisms could also block depolarization of the bacteria without affecting their viability, indicating that depolarization and death are not directly linked during HAMLET-induced death [20]. Still, the results with calcium and sodium inhibition suggested a certain level of parallel activation. Thus, we examined whether the same inhibitors could also inhibit these events induced by ELOA and OA*.*

**Figure 5:**
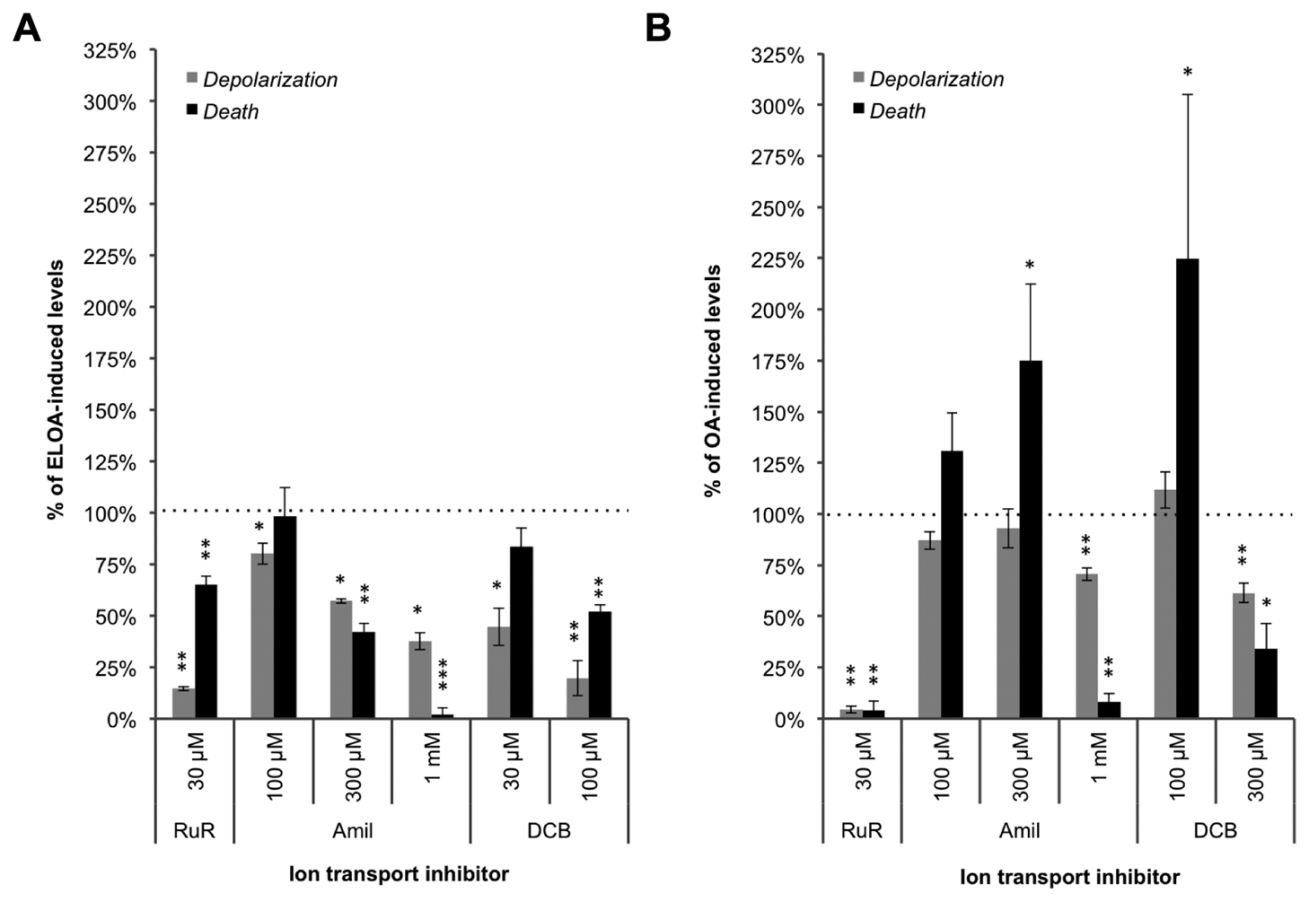
Inhibition of calcium channels and sodium-calcium exchange activity blocks bactericidal activity. D39∆*lytA* pneumococci were incubated with ELOA (40 µg/ml; **A**) or OA (32 µg/ml; **B**) for 1 h in the presence of Ruthenium Red (RuR), amiloride (Amil), or dichlorobenzamil (DCB), while monitoring membrane depolarization (DiBAC fluorescence). At the end of the incubation, the samples were serially diluted for viable counts to calculate logs of death. Depolarization (*gray bars*) and death (*black bars*) are expressed as a percentage of the values observed in the samples treated with ELOA alone or with OA alone (“ELOA alone” and “OA alone” values were set to 100%, represented by *dotted* *line*). Data represent the mean of three individual experiments, with S.D. *error bars*, and significance was calculated using the paired *t* test with a 95% confidence interval (* = *P* < 0.05; ** = *P* < 0.01; *** = *P < 0.001*).

The inhibitors alone did not produce any effect on the bacterial depolarization or viability (data not shown). We observed that RuR was able to very effectively inhibit the membrane depolarization triggered by ELOA, while also significantly rescuing the viability of the bacteria, suggesting an important role for the Ca^2+^ influx in the activation of ELOA-induced bacterial death (Figure **5A**). A concentration of ELOA (40 µg/ml) that produced 3-4 log_10_ death of pneumococci was used in these experiments to reliably enable measurements of inhibition. To examine the possibility that NCX activity is involved in the bactericidal mechanism, we first introduced the general Na^+^ channel inhibitor amiloride, which effectively blocked ELOA-induced bactericidal activity in a dose-dependent manner (Figure **5A**), with a 300 µM concentration required to block roughly half of the depolarization (*P* = 0.02) and log_10_ death (*P* = 0.002). We then introduced the amiloride derivative DCB, which has greater inhibitory specificity for Na^+^/Ca^2+^ exchangers, and observed that it also displayed very effective dose-dependent inhibition, but at much lower concentrations than amiloride, with 30 µM and 100 µM concentrations required to block more than half of the depolarization (*P* = 0.027) and log_10_ death (*P* = 0.003), respectively. This inhibition pattern is consistent with NCX activity and indicates that it is important for ELOA-induced death. The lack of a causal link between depolarization and bacterial death shown for HAMLET was similarly shown here as the inhibitors blocked the two events to different degrees (Figure **5A**). 

The results using these ion transport inhibitory compounds on the bactericidal activity of OA were phenotypically different (Figure **5B**). OA was used at twice the concentration present in the ELOA complex, a concentration that induced the same level of pneumococcal death as did ELOA above. However, inhibition with RuR was more effective for OA than for ELOA, resulting in nearly complete inhibition of both membrane depolarization (*P* = 0.007) and death (*P* = 0.002). On the other hand, introducing low concentrations of Amiloride and DCB caused significantly elevated levels of pneumococcal death, and only when high concentrations of these inhibitors were used, significant reduction in depolarization (amiloride *P* = 0.002; DCB *P* = 0.003) and rescue of the bacteria from death (amiloride *P* = 0.008; DCB *P* = 0.01) was observed (Figure **5B**). 

### ELOA and OA activate high molecular weight DNA fragmentation

**Figure 6:**
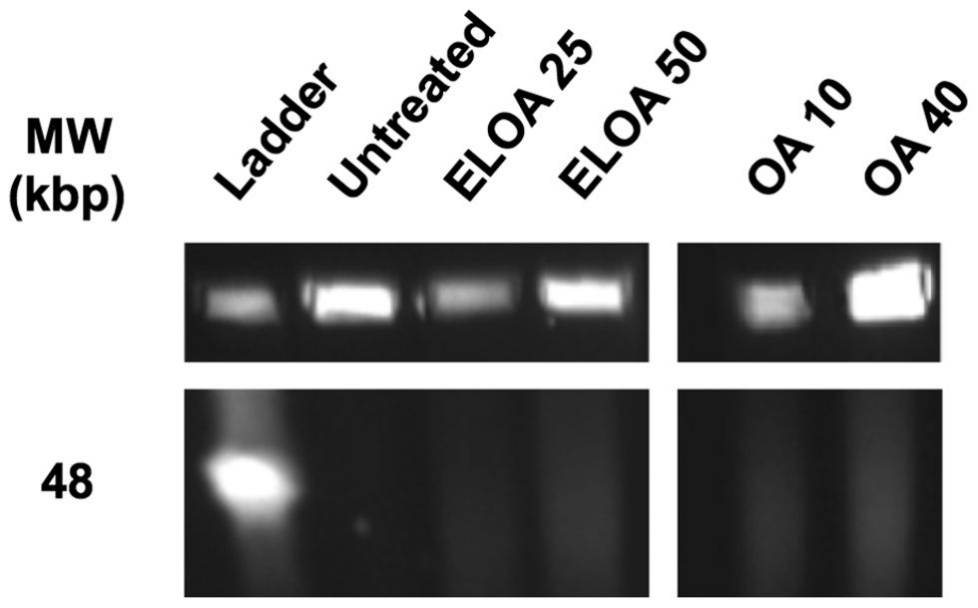
Pneumococcal DNA is fragmented into HMW sizes in ELOA- and OA-treated cells. Following treatment with ELOA or OA, DNA from D39 pneumococci was separated using pulse-field gel electrophoresis. OA samples were run on a separate gel. The number behind each indicated treatment refers to the concentration in µg/mL. The top panels represent intact chromosomal DNA and the lower panels the high molecular weight fragments obtained after treatment.

One of the most consistent hallmarks of eukaryotic apoptosis is the specific fragmentation of DNA, which is observed when chromatin is degraded into high molecular weight segments consisting of DNA rosettes and loops (50-300 kbp) [32], followed by the classical oligonucleosomal DNA fragmentation [33]. It has been shown previously that, like HAMLET, ELOA induces apoptotic cell death of eukaryote cells [11], so we investigated if bacterial death induced by ELOA was also accompanied by DNA fragmentation, as is observed during HAMLET-induced pneumococcal death [19]. Indeed, high molecular weight DNA fragments with sizes very similar to those detected in eukaryote apoptosis were observed in pneumococci treated with both ELOA and OA (Figure **6**), although the concentration of OA required for equal bacterial death and DNA fragmentation was consistently higher than the concentration of OA present in the ELOA complex (Figure **6**; *last two lanes*). These results suggest a similar phenotypic end-point morphology induced by ELOA and by OA downstream of activation of death at the membrane. 

## Discussion

In the present study we aimed to show that bactericidal activity can be a general property of protein-OA complexes similar to their tumoricidal and cytotoxic capacities. Although bactericidal activity has been reported for OA in complex with parvalbumin and beta-lactoglobulin [16], no comprehensive analysis of a complex besides HAMLET [19,20,34,35] has been conducted to date, and no molecular characterization of the bactericidal activities of protein-OA complexes compared with those of OA alone have been evaluated. For this purpose, we explored in detail the bactericidal activity of a second complex, consisting of equine lysozyme with oleic acid (ELOA) and compared it to the activity of OA alone and our earlier characterizations of the bactericidal activity of HAMLET [19,20]. ELOA was chosen based on the close homology of EL with ALA, but also based on the differences in HAMLET’s and ELOA’s functional properties as it relates to eukaryote cells. First, EL is the closest homolog to human and bovine alpha-lactalbumins with regards to sequences and tertiary structure, yet possesses different functionality (peptidoglycan hydrolysis versus lactose synthase activity, respectively) and folding properties [36]. Second, within ELOA, EL is present in a partially unfolded state, similar to ALA in HAMLET, though its molten globule differs drastically from the classical molten globules of alpha-lactalbumins [11]. Third, in contrast to HAMLET that can be either monomeric or oligomeric with low content of OA (5-8 OA molecules per protein molecule) [5], ELOA produced to date is always oligomeric and contains a rather high content of oleic acid (11 to 48 oleic acids per protein molecule) [11]. Finally, HAMLET has a tumor-specific activity, acting on many intracellular targets [4], whereas ELOA is indiscriminately toxic to all studied cells and acts at the level of the cell membrane to cause membrane defects, resulting in cell lysis and death [11,15,37]. 

We demonstrate here for the first time that ELOA possesses bactericidal activity against *S. pneumoniae*. Just like HAMLET, ELOA effectively killed pneumococci in a dose-dependent manner within the concentration range of 20-100 µg/ml (1 to 5 µM). The mechanism and phenotype of ELOA-induced death was shown to be similar to that induced by HAMLET when using lower ELOA-concentrations. As expected from interaction studies of HAMLET with artificial membranes [38,39], the ELOA complex accumulated at the pneumococcal cell membrane, leading to irreversible depolarization and subsequent rupture. Although depolarization *per se* does not seem to be directly associated with bacterial death, uptake of calcium through a sodium/calcium exchange mechanism appears to be central also to ELOA’s bactericidal activity at low concentrations. This effect on ion channel transport is not specific to bacteria but has also been observed in *Chara corallina* green algae cells treated with HAMLET and other complexes of human alpha-lactalbumin with oleic acid prepared as described previously [40,41]. We also detected apoptosis-like high molecular weight DNA fragmentation during ELOA-induced pneumococcal death, which resembles an apoptotic mechanism observed previously in eukaryotic cells treated with ELOA [11]. Thus, based on the present study and the most recent characterizations [20], the ELOA and HAMLET complexes appear to induce similar effects on the membrane, thereby inducing death of *S. pneumoniae* that results in apoptosis-like morphology. 

However, at high concentrations of ELOA in this study, depolarization and rupture of the membrane were temporally indistinguishable, possibly suggesting that depolarization is a result of rupture rather than the other way around, similar to the incorporation of ELOA in eukaryote membranes leading to membrane destabilization and lysis [15,37]. This is something we have not observed when treating bacteria with HAMLET [19,20]. Still, both the effects of HAMLET and ELOA were inhibited by calcium and sodium transport inhibitors, although the inhibition patterns were slightly different between the complexes. RuR inhibited HAMLET-induced death more effectively than ELOA-induced death, whereas Amiloride inhibited HAMLET-induced death less effectively than ELOA-induced death (Figure 5A and [20]). This difference may represent both a difference in the protein moiety and the lipid to protein ratio of the complexes. 

Interestingly, complexes of OA with other proteins, such as parvalbumin and beta-lactoglobulin, have also recently been shown to exert bactericidal activities similar to HAMLET and ELOA [12,16]. This indicates, as has been proposed by others for both eukaryote and prokaryote cells [13-16], that OA plays a critical role in the cytotoxic and bactericidal activity of such complexes. Although OA has long been known to possess antibacterial activity, especially against gram-positive organisms [27-30,42], the specific mechanism of OA-induced bacterial death is not well defined [43]. Studies have shown that OA interferes with electron transfer and oxidative phosphorylation during aerobic respiration in species such as those of the genus *Bacillus* [44], and that increased membrane fluidity after OA-exposure can increase the permeability of protons, dissipating the proton motive force [45]. 

We therefore attempted to better understand the mechanism of OA-induced pneumococcal death to elucidate the bactericidal role of OA in protein-lipid complexes. Although this study provided evidence for similarities in the initial membrane effects required for ELOA and OA to induce bacterial death, the results indicated a number of differences. Although OA was shown to have cytotoxic activity against pneumococci, which has not been described in the literature, it had different kinetics and inhibition patterns when compared to ELOA. Significantly higher concentrations of OA than those present in the ELOA complex were required to confer the same level of bactericidal activity. Additionally, similar to ELOA-induced death, OA induced a calcium influx that could be blocked by addition of the calcium transport inhibitor RuR and sodium and sodium/calcium exchange inhibitors, but where ELOA showed a similar inhibition pattern as HAMLET, the bactericidal activity of OA was almost completely inhibited by RuR and significantly less so by the sodium transport inhibitors. Interestingly, low concentrations of sodium transport inhibitor significantly increased the depolarization and death with OA, which was not seen when adding ELOA or HAMLET. We can only speculate on these differences at this time and further studies are required to address the mechanisms. For RuR, besides its well documented ability to block calcium transport [46-49], it has also been shown to interact with and stabilize the cell wall and the lipid bilayer through low affinity binding [50,51]. OA alone may therefore be less effective in disrupting the membrane to cause ion transport. In contrast, OA being part of a protein complex may be delivered more selectively with the help of the protein to specific membrane domains, as has been seen in eukaryotic cells [37], potentially so that higher concentration of the RuR would be required to inhibit OA’s action. This weaker activation of ion transport is supported by the observation that OA-induced depolarization and rupture of the bacterial membrane was delayed in comparison to those same events induced by ELOA. For sodium inhibition, sodium transporters are ubiquitous throughout the membrane and inhibition of sodium transport may well destabilize the ion equilibrium and the membrane stability, where low concentrations of inhibitor increase the sensitivity to OA that targets the membrane in a general way, whereas the effect on ELOA that interacts in a targeted way with the membrane is going to be minimal at these concentrations. At higher concentrations, specific inhibition of sodium/calcium exchange activity activated by both OA and ELOA will override the initial increased sensitivity that is saturated at lower concentrations. 

Additionally, both ELOA and HAMLET forms annular oligomeric assemblies [11,52], similar to pore-like structures, produced by amyloid, viral proteins, or killing agents such as Bax and Bak that are involved in cytotoxicity [53-55]. It can therefore not be excluded that ELOA pores assembled within the membrane in localized areas may co-interact with the calcium and sodium channels and exacerbate the leak of corresponding ions. The oleic acid alone does not possess the equal capacity, which may also explain the different effects of inhibitors on the membrane permeability and cell death caused by ELOA and oleic acid. 

Overall, these results indicate that one function of the protein moiety in ELOA and in other complexes may be to solubilize the lipid better for presentation and targeting to specific domains or molecules in the bacterial membrane, where activity of the lipid can be more efficiently enacted compared to when OA is added by itself. Additionally, it is likely that the protein moiety of this family of protein-lipid complexes has effects of its own that can add to or synergize with the activity of the lipid and alter sensitivity to ion transport inhibition. The specificity of such interactions will, of course, depend on the protein involved, as well as the oligomerization state and oleic acid content in each complex, and may be one reason that the specificity of protein-lipid complexes against tumor cells versus healthy cells varies [8-14]. For example, complexes of OA with EL, parvalbumin, or beta-lactoglobulin appear to be much more toxic to healthy cells than OA in complex with human alpha-lactalbumin (HAMLET). We know that at least ELOA act directly on the membrane, whereas HAMLET act primarily through interaction with intracellular targets that HAMLET cannot reach in healthy cells. This targeting is likely due to the protein moiety of the complex [4,11,16], but could also be a result of the higher OA association in the ELOA complex. Similar to HAMLET’s tumoricidal activity [4,56] we have preliminary data suggesting that in addition to acting on the bacterial membrane, HAMLET also has intracellular targets in bacteria, such as inhibiting glycolytic enzymes (APH, unpublished observation). 

Moreover, complex formation with other related proteins such as hen egg lysozyme does not produce cytotoxic complexes (Wilhelm and Morozova-Roche, unpublished observation), suggesting that only certain proteins can form complexes with OA that possesses cytotoxic and bactericidal activities. Similarly, the methods of producing these complexes are known to result in complexes with various protein:lipid stoichiometry, different oligomeric states, and various free lipid content association, which may also be relevant for specificity and targeting of the complex [17].

In contrast to parvalbumin and beta-lactoglobulin, EL and human alpha-lactalbumin are both found in high content in horse and human milk, respectively, as is OA, which may be indicative that, apart from their anti-tumor activity, both complexes also carry out bactericidal function in the milk. Fermented horse milk “kumis” is well known for its remarkable bactericidal and therapeutic properties and has been very widely used in traditional medicine in Asia for centuries [57]. Thus, it is plausible that the abundant levels of EL and OA in the horse milk could complex and contribute to the therapeutic properties of “kumis”.

In summary, the ELOA complex, which can be easily formed from two naturally occurring and abundant components (EL and OA), exhibits similar bactericidal activity as HAMLET at lower concentrations, and shows differences in its pattern of activity compared with OA. ELOA’s potent bactericidal activity has the potential to be a novel tool in treating *S. pneumoniae* infection, with little risk for side-effects. Additionally, as the pathways that these protein-lipid complexes activate are further characterized and elucidated, the targets that are revealed have the potential to become attractive candidates in the quest for novel antimicrobial and antitumor drug targets.
